# Female chromosome X mosaicism is age-related and preferentially affects the inactivated X chromosome

**DOI:** 10.1038/ncomms11843

**Published:** 2016-06-13

**Authors:** Mitchell J. Machiela, Weiyin Zhou, Eric Karlins, Joshua N. Sampson, Neal D. Freedman, Qi Yang, Belynda Hicks, Casey Dagnall, Christopher Hautman, Kevin B. Jacobs, Christian C. Abnet, Melinda C. Aldrich, Christopher Amos, Laufey T. Amundadottir, Alan A. Arslan, Laura E. Beane-Freeman, Sonja I. Berndt, Amanda Black, William J. Blot, Cathryn H. Bock, Paige M. Bracci, Louise A. Brinton, H Bas Bueno-de-Mesquita, Laurie Burdett, Julie E. Buring, Mary A. Butler, Federico Canzian, Tania Carreón, Kari G. Chaffee, I-Shou Chang, Nilanjan Chatterjee, Chu Chen, Constance Chen, Kexin Chen, Charles C. Chung, Linda S. Cook, Marta Crous Bou, Michael Cullen, Faith G. Davis, Immaculata De Vivo, Ti Ding, Jennifer Doherty, Eric J. Duell, Caroline G. Epstein, Jin-Hu Fan, Jonine D. Figueroa, Joseph F. Fraumeni, Christine M. Friedenreich, Charles S. Fuchs, Steven Gallinger, Yu-Tang Gao, Susan M. Gapstur, Montserrat Garcia-Closas, Mia M. Gaudet, J. Michael Gaziano, Graham G. Giles, Elizabeth M. Gillanders, Edward L. Giovannucci, Lynn Goldin, Alisa M. Goldstein, Christopher A. Haiman, Goran Hallmans, Susan E. Hankinson, Curtis C. Harris, Roger Henriksson, Elizabeth A. Holly, Yun-Chul Hong, Robert N. Hoover, Chao A. Hsiung, Nan Hu, Wei Hu, David J. Hunter, Amy Hutchinson, Mazda Jenab, Christoffer Johansen, Kay-Tee Khaw, Hee Nam Kim, Yeul Hong Kim, Young Tae Kim, Alison P. Klein, Robert Klein, Woon-Puay Koh, Laurence N. Kolonel, Charles Kooperberg, Peter Kraft, Vittorio Krogh, Robert C. Kurtz, Andrea LaCroix, Qing Lan, Maria Teresa Landi, Loic Le Marchand, Donghui Li, Xiaolin Liang, Linda M. Liao, Dongxin Lin, Jianjun Liu, Jolanta Lissowska, Lingeng Lu, Anthony M. Magliocco, Nuria Malats, Keitaro Matsuo, Lorna H. McNeill, Robert R. McWilliams, Beatrice S. Melin, Lisa Mirabello, Lee Moore, Sara H. Olson, Irene Orlow, Jae Yong Park, Ana Patiño-Garcia, Beata Peplonska, Ulrike Peters, Gloria M. Petersen, Loreall Pooler, Jennifer Prescott, Ludmila Prokunina-Olsson, Mark P. Purdue, You-Lin Qiao, Preetha Rajaraman, Francisco X. Real, Elio Riboli, Harvey A. Risch, Benjamin Rodriguez-Santiago, Avima M. Ruder, Sharon A. Savage, Fredrick Schumacher, Ann G. Schwartz, Kendra L. Schwartz, Adeline Seow, Veronica Wendy Setiawan, Gianluca Severi, Hongbing Shen, Xin Sheng, Min-Ho Shin, Xiao-Ou Shu, Debra T. Silverman, Margaret R. Spitz, Victoria L. Stevens, Rachael Stolzenberg-Solomon, Daniel Stram, Ze-Zhong Tang, Philip R. Taylor, Lauren R. Teras, Geoffrey S. Tobias, David Van Den Berg, Kala Visvanathan, Sholom Wacholder, Jiu-Cun Wang, Zhaoming Wang, Nicolas Wentzensen, William Wheeler, Emily White, John K. Wiencke, Brian M. Wolpin, Maria Pik Wong, Chen Wu, Tangchun Wu, Xifeng Wu, Yi-Long Wu, Jay S. Wunder, Lucy Xia, Hannah P. Yang, Pan-Chyr Yang, Kai Yu, Krista A. Zanetti, Anne Zeleniuch-Jacquotte, Wei Zheng, Baosen Zhou, Regina G. Ziegler, Luis A. Perez-Jurado, Neil E. Caporaso, Nathaniel Rothman, Margaret Tucker, Michael C. Dean, Meredith Yeager, Stephen J. Chanock

**Affiliations:** 1 Division of Cancer Epidemiology and Genetics, National Cancer Institute (NCI), National Institutes of Health (NIH), Bethesda, Maryland 20892, USA; 2 Cancer Genomics Research Laboratory, National Cancer Institute, Division of Cancer Epidemiology and Genetics, Leidos Biomedical Research Inc., Bethesda, Maryland 20892, USA; 3 Bioinformed, LLC, Gaithersburg, Maryland 20877, USA; 4 Department of Thoracic Surgery, Vanderbilt University School of Medicine, Nashville, Tennessee 37232, USA; 5 Division of Epidemiology, Department of Medicine, Vanderbilt Epidemiology Center, Vanderbilt University Medical Center, Nashville, Tennessee 37232, USA; 6 Department of Epidemiology, Division of Cancer Prevention and Population Sciences, The University of Texas MD Anderson Cancer Center, Houston, Texas 77030, USA; 7 Department of Obstetrics and Gynecology, New York University School of Medicine, New York, New York 10016, USA; 8 Department of Environmental Medicine, New York University School of Medicine, New York, New York 10016, USA; 9 New York University Cancer Institute, New York, New York 10016, USA; 10 International Epidemiology Institute, Rockville, Maryland 20850, USA; 11 Karmanos Cancer Institute and Department of Oncology, Wayne State University School of Medicine, Detroit, Michigan 48201, USA; 12 Department of Epidemiology and Biostatistics, University of California San Francisco, San Francisco, California 94143, USA; 13 Department for Determinants of Chronic Diseases (DCD), National Institute for Public Health and the Environment (RIVM), 3721 Bilthoven, The Netherlands; 14 Department of Gastroenterology and Hepatology, University Medical Center, 3584 CX Utrecht, The Netherlands; 15 Department of Epidemiology and Biostatistics, The School of Public Health, Imperial College London, London SW7 2AZ, UK; 16 Department of Social and Preventive Medicine, Faculty of Medicine, University of Malaya, Kuala Lumpur 50603, Malaysia; 17 Division of Preventive Medicine, Brigham and Women's Hospital, Boston, Massachusetts 02115, USA; 18 National Institute for Occupational Safety and Health, Centers for Disease Control and Prevention, Cincinnati, Ohio 45226, USA; 19 Division of Cancer Epidemiology, German Cancer Research Center (DKFZ), 69120 Heidelberg, Germany; 20 Department of Health Sciences Research, Mayo Clinic, Rochester, Minnesota 55905, USA; 21 National Institute of Cancer Research, National Health Research Institutes, Zhunan 35053, Taiwan; 22 Division of Public Health Sciences, Fred Hutchinson Cancer Research Center, Seattle, Washington 98109, USA; 23 Program in Genetic Epidemiology and Statistical Genetics, Harvard School of Public Health, Boston, Massachusetts 02115, USA; 24 Department of Epidemiology and Biostatistics, Tianjin Medical University Cancer Institute and Hospital, Tianjin 300040, China; 25 University of New Mexico, Albuquerque, New Mexico 87131, USA; 26 Channing Division of Network Medicine, Department of Medicine, Brigham and Women's Hospital and Harvard Medical School, Boston, Massachusetts 02115, USA; 27 Department of Public Health Sciences, School of Public Health, University of Alberta, Edmonton, Alberta, Canada T6G 2R3; 28 Shanxi Cancer Hospital, Taiyuan, Shanxi 030013, China; 29 Geisel School of Medicine, Dartmouth College, Lebanon, New Hampshire 03755, USA; 30 Unit of Nutrition and Cancer, Cancer Epidemiology Research Program, Bellvitge Biomedical Research Institute, Catalan Institute of Oncology (ICO-IDIBELL), 08908 Barcelona, Spain; 31 Shanghai Cancer Institute, Shanghai 200032, China; 32 Department of Population Health Research, Cancer Control Alberta, Alberta Health Services, Calgary, Alberta, Canada T2N 2T9; 33 Department of Medical Oncology, Dana-Farber Cancer Institute, Boston, Massachusetts 02215, USA; 34 Fred A Litwin Centre for Cancer Genetics, Samuel Lunenfeld Research Institute, Toronto, Ontario, Canada M5G 1X5; 35 Department of Epidemiology, Shanghai Cancer Institute, Renji Hospital, Shanghai Jiaotaong University School of Medicine, Shanghai 200032, China; 36 Epidemiology Research Program, American Cancer Society, Atlanta, Georgia 30303, USA; 37 Division of Genetics and Epidemiology, and Breakthrough Breast Cancer Centre, Institute for Cancer Research, London SM2 5NG, UK; 38 Divisions of Preventive Medicine and Aging, Department of Medicine, Brigham and Women's Hospital and Harvard Medical School, Boston, Massachusetts 02115, USA; 39 Massachusetts Veterans Epidemiology Research and Information Center/VA Cooperative Studies Programs, Veterans Affairs Boston Healthcare System, Boston, Massachusetts 02130, USA; 40 Cancer Epidemiology Centre, Cancer Council Victoria & Centre for Epidemiology and Biostatistics, Melbourne School of Population and Global Health, The University of Melbourne, Melbourne, Victoria 3010, Australia; 41 Division of Cancer Control and Population Sciences, National Cancer Institute, Bethesda, Maryland 20892, USA; 42 Department of Epidemiology, Harvard School of Public Health, Boston, Massachusetts 02115, USA; 43 Department of Preventive Medicine, Biostatistics Division, Keck School of Medicine at the University of Southern California, Los Angeles, California 90033, USA; 44 Department of Public Health and Clinical Medicine/Nutritional Research, Umeå University, 901 87 Umeå, Sweden; 45 Division of Biostatistics and Epidemiology, University of Massachusetts School of Public Health and Health Sciences, Amherst, Massachusetts 01003, USA; 46 Laboratory of Human Carcinogenesis, Center for Cancer Research, National Cancer Institute, Bethesda, Maryland 20892, USA; 47 Department of Radiation Sciences, Oncology, Umeå University, 901 87 Umeå, Sweden; 48 Department of Preventive Medicine, Seoul National University College of Medicine, Seoul 151-742, Republic of Korea; 49 Institute of Population Health Sciences, National Health Research Institutes, Zhunan 35053, Taiwan; 50 Broad Institute of Harvard and MIT, Cambridge, Massachusetts 02142, USA; 51 International Agency for Research on Cancer (IARC-WHO), 69372 Lyon, France; 52 Oncology, Finsen Centre, Rigshospitalet, 2100 Copenhagen, Denmark; 53 Unit of Survivorship Research, The Danish Cancer Society Research Centre, 2100 Copenhagen, Denmark; 54 School of Clinical Medicine, University of Cambridge, Cambridge CB2 1TN, UK; 55 Center for Creative Biomedical Scientists, Chonnam National University, Gwangju 500-757, Republic of Korea; 56 Department of Internal Medicine, Division of Oncology/Hematology, College of Medicine, Korea University Anam Hospital, Seoul 151-742, Republic of Korea; 57 Department of Thoracic and Cardiovascular Surgery, Cancer Research Institute, Seoul National University College of Medicine, Seoul 03080, Republic of Korea; 58 Department of Oncology, The Johns Hopkins University School of Medicine, Baltimore, Maryland 21287, USA; 59 Program in Cancer Biology and Genetics, Memorial Sloan-Kettering Cancer Center, New York, New York, 10065, USA; 60 Duke-NUS Graduate Medical School, Singapore 169857, Singapore; 61 Saw Swee Hock School of Public Health, National University of Singapore, Singapore 119077, Singapore; 62 Epidemiology Program, University of Hawaii Cancer Center, Honolulu, Hawaii 96813, USA; 63 Fondazione IRCCS Istituto Nazionale dei Tumori, Milano 20133, Italy; 64 Department of Medicine, Memorial Sloan-Kettering Cancer Center, New York, New York 10065, USA; 65 Department of Gastrointestinal Medical Oncology, The University of Texas MD Anderson Cancer Center, Houston, Texas 77030, USA; 66 Memorial Sloan-Kettering Cancer Center, New York, New York 10065, USA; 67 Department of Etiology & Carcinogenesis, Cancer Institute and Hospital, Chinese Academy of Medical Sciences and Peking Union Medical College, Beijing 100730, China; 68 State Key Laboratory of Molecular Oncology, Cancer Institute and Hospital, Chinese Academy of Medical Sciences and Peking Union Medical College, Beijing 100730, China; 69 Department of Human Genetics, Genome Institute of Singapore 138672, Singapore; 70 School of Life Sciences, Anhui Medical University, Hefei 230032, China; 71 Department of Cancer Epidemiology and Prevention, Maria Sklodowska-Curie Cancer Center and Institute of Oncology, Warsaw 02-781, Poland; 72 Yale School of Public Health, New Haven, Connecticut 06510, USA; 73 H. Lee Moffitt Cancer Center and Research Institute, Tampa, Florida 33612, USA; 74 Spanish National Cancer Research Centre (CNIO), Madrid 28029, Spain; 75 Division of Molecular Medicine, Aichi Cancer Center Research Institute, Nagoya 464-8681, Japan; 76 Department of Health Disparities Research, Division of OVP, Cancer Prevention and Population Sciences, and Center for Community-Engaged Translational Research, Duncan Family Institute, The University of Texas MD Anderson Cancer Center, Houston, Texas 77030, USA; 77 Department of Oncology, Mayo Clinic, Rochester, Minnesota 55905, USA; 78 Lung Cancer Center, Kyungpook National University Medical Center, Daegu 101, Republic of Korea; 79 Department of Pediatrics, University Clinic of Navarra, Universidad de Navarra, IdiSNA, Navarra Institute for Health Research, Pamplona 31080, Spain; 80 Nofer Institute of Occupational Medicine, Lodz 91-348, Poland; 81 University of Southern California, Los Angeles, California 90007, USA; 82 Department of Epidemiology, Cancer Institute (Hospital), Chinese Academy of Medical Sciences, Beijing 100730, China; 83 Departament de Ciències Experimentals i de la Salut, Universitat Pompeu Fabra, Barcelona 08002, Spain; 84 Division of Epidemiology and Biostatistics, School of Public Health, Imperial College London, London SW7 2AZ, UK; 85 Centro de Investigación Biomédica en Red de Enfermedades Raras (CIBERER), Barcelona, 28029, Spain; 86 Quantitative Genomic Medicine Laboratory, qGenomics, Barcelona 08003, Spain; 87 Karmanos Cancer Institute and Department of Family Medicine and Public Health Sciences, Wayne State University School of Medicine, Detroit, Michigan 48201, USA; 88 Human Genetics Foundation (HuGeF), Torino, 10126, Italy; 89 Jiangsu Key Laboratory of Cancer Biomarkers, Prevention and Treatment, Nanjing Medical University, Nanjing 210029, China; 90 Department of Epidemiology, Nanjing Medical University School of Public Health, Nanjing 210029, China; 91 Department of Preventive Medicine, Chonnam National University Medical School, Gwanju 501-746, Republic of Korea; 92 Department of Medicine, Vanderbilt Epidemiology Center, Vanderbilt-Ingram Cancer Center, Vanderbilt University Medical Center, Nashville, Tennessee 37232, USA; 93 Baylor College of Medicine, Houston, Texas 77030, USA; 94 Johns Hopkins Bloomberg School of Public Health, Baltimore, Maryland 21218, USA; 95 Ministry of Education Key Laboratory of Contemporary Anthropology, School of Life Sciences, Fudan University, Shanghai 200433, China; 96 State Key Laboratory of Genetic Engineering, School of Life Sciences, Fudan University, Shanghai 200433, China; 97 Information Management Services Inc., Calverton, Maryland, 20904, USA; 98 University of California San Francisco, San Francisco, California 94143, USA; 99 Department of Pathology, Li Ka Shing Faculty of Medicine, The University of Hong Kong, Hong Kong, China; 100 Institute of Occupational Medicine and Ministry of Education Key Laboratory for Environment and Health, School of Public Health, Huazhong University of Science and Technology, Wuhan 430400, China; 101 Department of Epidemiology, The University of Texas MD Anderson Cancer Center, Houston, Texas 77030, USA; 102 Guangdong Lung Cancer Institute, Guangdong General Hospital & Guangdong Academy of Medical Sciences, Guangzhou 515200, China; 103 Division of Urologic Surgery, Washington University School of Medicine, St Louis, Missouri 63110, USA; 104 Department of Internal Medicine, National Taiwan University College of Medicine, Taipei 10617, Taiwan; 105 Department of Population Health, New York University School of Medicine, New York, New York 10016, USA; 106 Department of Epidemiology, School of Public Health, China Medical University, Shenyang 110001, China

## Abstract

To investigate large structural clonal mosaicism of chromosome X, we analysed the SNP microarray intensity data of 38,303 women from cancer genome-wide association studies (20,878 cases and 17,425 controls) and detected 124 mosaic X events >2 Mb in 97 (0.25%) women. Here we show rates for X-chromosome mosaicism are four times higher than mean autosomal rates; X mosaic events more often include the entire chromosome and participants with X events more likely harbour autosomal mosaic events. X mosaicism frequency increases with age (0.11% in 50-year olds; 0.45% in 75-year olds), as reported for Y and autosomes. Methylation array analyses of 33 women with X mosaicism indicate events preferentially involve the inactive X chromosome. Our results provide further evidence that the sex chromosomes undergo mosaic events more frequently than autosomes, which could have implications for understanding the underlying mechanisms of mosaic events and their possible contribution to risk for chronic diseases.

## Abstract (web summary)

It is unclear how often genetic mosaicism of chromosome X arises. Here, the authors examine women with cancer and cancer-free controls and show that X chromosome mosaicism occurs more frequently than on autosomes, especially on the inactive X chromosome, but is not linked to non-haematologic cancer risk

## Graphical abstract

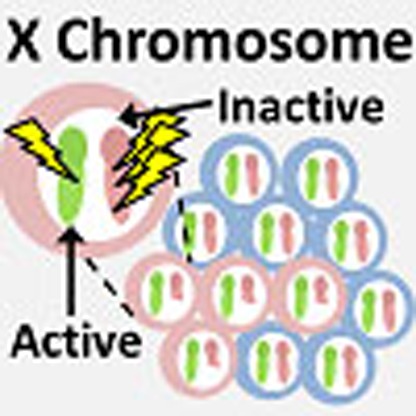

Genetic mosaicism is classically defined as the coexistence of clonal cellular populations harbouring two or more distinct genotypes^1^. To date, detectable mosaicism has been reported in apparently healthy individuals as well as in patients with rare diseases, such as neurofibromatosis type II (NF2), trisomy 21, naevus sebaceous and Proteus syndrome^2^,^3^,^4^,^5^,^6^,^7^,^8^,^9^. Emerging data from consortia of genome-wide association studies (GWAS)^3^,^5^,^6^,^10^,^11^,^12^ have demonstrated large autosomal mosaicism (events >2 Mb in size) in DNA collected from peripheral leukocytes and buccal epithelium. These studies suggest that autosomal mosaicism is associated with aging, hematologic cancer risk, and possibly ancestry and male sex. Whereas autosomal mosaicism is detectable in <2% of older individuals, recent studies indicate that large mosaic events may be far more common for the Y chromosome, and in particular among older men who smoke cigarettes^13^,^14^,^15^.

The functional consequences of detectable chromosomal mosaicism remain to be fully determined. A number of groups have reported detectable genetic mosaicism of single-nucleotide mutations in the general population, particularly in genes implicated in hematopoietic disorders such as leukaemias and lymphomas^2^,^4^,^16^. Point-mutation events could reflect early, preleukemic clones and separately could increase risk for cardiovascular events^4^. Moreover, many reports have shown phenotypic consequences of chromosomal mosaicism that vary by genomic location of the event, developmental timing, tissue type involved and percentage of cells affected^7^,^8^,^9^. In prospective cohort studies, it has been possible to detect large mosaic structural events in blood samples of individuals who eventually develop chronic leukaemia, as early as 14 years before diagnosis, suggesting detection of a subset of events that eventually become manifest as part of the molecular profile of leukaemia^3^,^5^,^17^.

To date, reports have not systematically addressed the frequency and characteristics of X chromosomal mosaicism. The X chromosome is unique among the human chromosomes in that normal women carry two copies and normal men carry one. To compensate for dosage differences between sexes, one copy of the female X chromosome is rendered transcriptionally inactive in a process called X inactivation^18^. In humans, the inactive X-chromosome (Xi) is randomly chosen early in development. Once established, X inactivation is generally irreversible and stably maintained through mitotic divisions. Established mechanisms for maintaining X inactivation include expression of the non-coding *XIST* RNA, chromatin modifications, changes in nuclear scaffold proteins, and DNA methylation^19^,^20^,^21^,^22^,^23^. Sequence data from cancer genomes suggest that the X chromosome, particularly the female Xi, has a higher somatic mutation load of point mutations than the autosomes^24^. It has been postulated that the observed higher load of somatic point mutations could be directly related to the timing of Xi replication, which occurs late and is faster than either the active X-chromosome (Xa) or the autosomes^25^,^26^,^27^. Although these and other data suggest that X-chromosome mosaicism may be detectable at a prevalence higher than that observed on the autosomes^28^,^29^,^30^, little is known about its frequency in the population or basic characteristics of the distribution and types of gains, losses and acquired loss of heterozygosity.

In this report, we investigate the frequency of large-scale chromosome X mosaicism (>2 Mb) in blood or buccal samples from 38,303 women. We observe an overall frequency of X mosaicism of ∼0.25%, roughly four times the mean autosomal rate. The frequency of X mosaicism increases with increasing age, but is not associated with non-haematologic cancer risk. Further investigations by methylation analyses suggest the inactive X chromosome is preferentially gained or lost in X mosaic events.

## Results

### Detected chromosome X events

**Figure 1:**
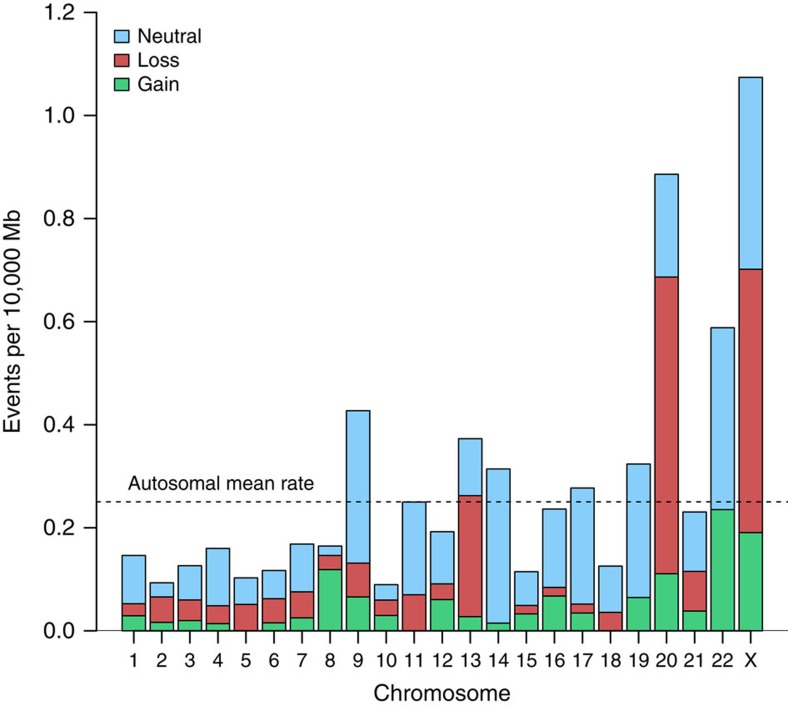
Adjusted mean rate of events by chromosome. A comparison of detected mosaic events >2 Mb in size in the autosomes to the X chromosome (X events=124, Autosomal events=430).

**Figure 2:**
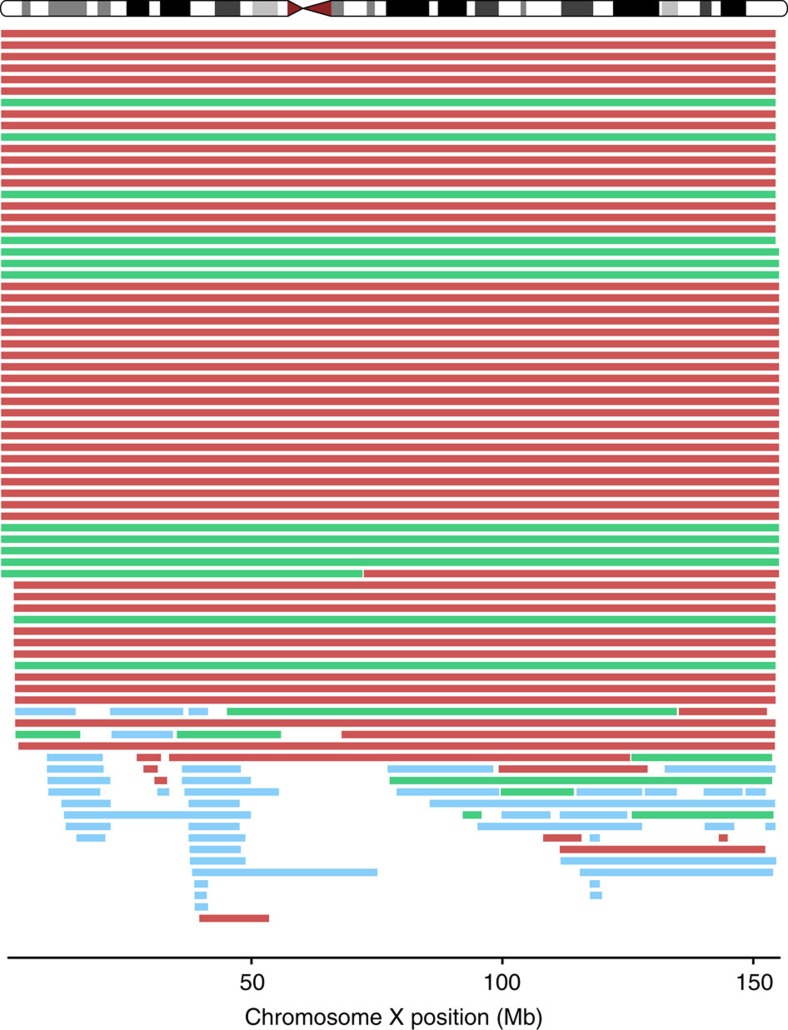
Detected mosaic events on the X chromosome. Mosaic losses (*N*=59) are in red, mosaic gains (*N*=22) are green, and mosaic copy-neutral events (*N*=43) are in blue.

Using a segmentation algorithm, we conducted a systematic scan of large structural detectable mosaicism on the X chromosomes of 38,303 women (20,878 cancer cases and 17,425 cancer-free controls), who had been previously examined for autosomal mosaicism^3^,^11^,^12^. In total, 124 mosaic events greater than 2 Mb in size were detected on the X chromosomes of 97 of the 38,303 women who were scanned (0.25%, Supplementary Table 1, Supplementary Table 2); all detected cases of trisomy X and XO (Turner's syndrome) were removed from subsequent analyses (*n*=5). Of the 97 women with detected X events, 15 (15%) had more than one event detected on their X chromosome, with one woman having as many as five events. The base-pair adjusted rate of mosaic X events was 1.07 events per 10,000 Mb, over fourfold higher than the mean 0.25 events per 10,000 Mb rate observed across the autosomes^12^ (*P* value=1.32 × 10^−5^, Fig. 1). Significantly elevated rates were observed for the X chromosome in comparison with all autosomes except for chromosome 20 (chr20=0.89, chrX=1.07 events per 10,000 MB; *P* value=0.29). The 124 mosaic X events consisted of 59 mosaic losses, 43 mosaic copy-neutral events and 22 mosaic gains (Fig. 2, Supplementary Fig. 1). These events mostly included the whole chromosome, with a fraction (37%) mapping to the interstitial region (Table 1). Few events were found at either the centromeric or telomeric ends. Most whole-X-chromosome events were mosaic losses. Interstitial events were primarily mosaic copy-neutral loss of heterozygosity, which have been less extensively documented in the cytogenetic literature on chromosome X (Supplementary Table 3). Two notable clusters of interstitial mosaic copy-neutral events are centered at approximately 26 and 49 Mb (NCBI36/hg18, Fig. 2). While X-chromosome mosaic events were more common than autosomal events, the mean proportion of cells with X-chromosome mosaicism tended to be lower than the mosaic proportion with autosomal events overall (X=0.299, autosomes=0. 359, *P* value=0.01, Supplementary Fig. 2), however, this association was not observed in cancer-free individuals (*P* value=0.10). Women with an X-chromosome mosaic event had a significantly higher likelihood of harbouring an autosomal event relative to women without detectable X mosaicism (unadjusted odds ratio (OR_unadj_)=16.7, 95% confidence interval (CI)=8.3-33.6, *P* value=2.5 × 10^−15^), even after adjusting for age (adjusted odds ratio (OR_adj_)=15.6, 95% CI=7.3-33.0, *P* value=8.6 × 10^−13^).

### Validation by qPCR

Detected X mosaic events were experimentally validated using a set of 12 quantitative PCR assays (qPCR) across chromosome X. Specifically, we estimated copy-number ratios for 26 events across 25 females with single-nucleotide polymorphism (SNP) microarray-detected X mosaicism with a range of mosaic proportions from 6 to 88%. In the 18 mosaic samples with events that spanned the entire X chromosome, the concordance rate was 100% for gains and 80% for losses (Supplementary Table 5). An inspection of the discordant copy-loss samples called as copy-neutral events revealed qPCR copy-number values near the calling threshold, or samples with low mosaic proportions. For detected mosaic events spanning only a portion of the X chromosome, four of the eight (50%) showed evidence for mosaic copy-number changes by qPCR, although only 25% were concordant in copy-number state with qPCR (Supplementary Table 5), suggesting the limited subsets of qPCR probes that spanned events may have been insufficient to adequately call copy-number states.

### X mosaicism in men

We also examined X-chromosome mosaicism in men. Although we identified 187 men with suggestive evidence of X-chromosome mosaicism (from 43,735 scanned participants), results from qPCR validation in 39 men with available DNA were poor (15% concordance). Calling X-chromosome mosaicism is inherently more challenging in men as their possession of a single X-chromosome precludes analysis with the B-allele frequency (BAF). Although certainly of interest, further refinement of the calling algorithm is required before we can reliably call detectable X mosaicism in men. All subsequent analyses of X mosaicism reported herein are restricted to women.

### X mosaicism associations

**Figure 3:**
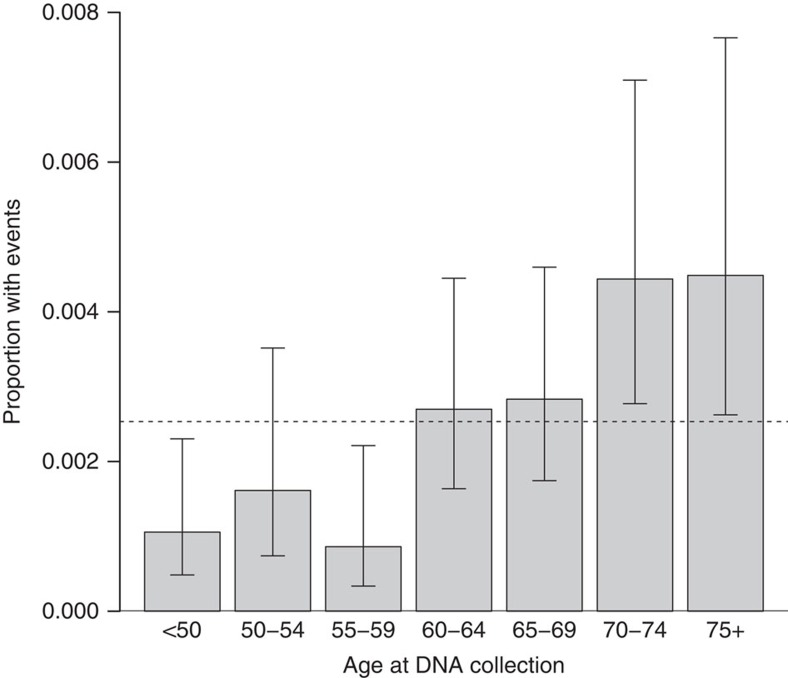
Unadjusted age relationship with X mosaicism. Dashed line represents the mean overall proportion with mosaic X events across all age groups and error pars represent 95% Wilson confidence intervals (*N*=31,982).

Detectable X mosaicism increases with age, with more events in older women than in younger women. The estimated frequency of X mosaicism was 0.11% in women under 50 years of age and 0.45% in women 75 years or older (Fig. 3). Multivariate analyses adjusted for ancestry, cancer status and study found a statistically significant association with an OR of 1.04 per 1-year increase in age (95% CI=1.01–1.06, *P* value=0.005), with a 20-year increase in age resulting in over twice the odds of a acquiring a mosaic event on the X chromosome. Altogether with prior evidence from autosomes and the Y chromosome^12^,^13^, our data suggest that each human chromosome is susceptible to age-related structural deterioration related to clonal mosaicism, but at distinct rates. Y mosaic events are more common than X events, and X events are more common than those in autosomes. These frequencies may reflect intrinsic differences in the mechanisms by which each type of chromosome is replicated or protected against age-related DNA damage^26^.

Comparable to what we reported for the autosomes (in over 127,000 individuals scanned), we found little to no evidence for an overall association between X mosaicism and non-haematologic cancer (*P* value=0.19)^3^,^5^,^12^. An analysis by cancer site found at most a marginally significant association between X mosaicism and lung cancer risk (OR=1.89, 95% CI=1.02–3.50, *P* value=0.042; 26 lung cancer cases with mosaicism). However, we had only a limited sample size, were unable to adequately adjust for the major lung cancer risk factor, cigarette smoking and we did not consider multiple comparisons across cancer types. We did not detect an association between X mosaicism and ancestry (three continental populations: European, African and East Asian, *P* value=0.40) that was detected in prior autosomal mosaicism analyses^12^. In addition, we did not find evidence for an association between X mosaicism and smoking for a subset of women with available smoking information (*N*=19,197, ever smoker versus never smoker *P* value=0.54). Interestingly, an association was found between DNA source and X mosaicism in which X mosaicism was more frequent in buccal cells as compared with leukocytes (OR=3.50, 95% CI=1.45–8.46, *P* value=0.005). Larger studies are required to confirm these findings.

### Methylation analysis

**Figure 4:**
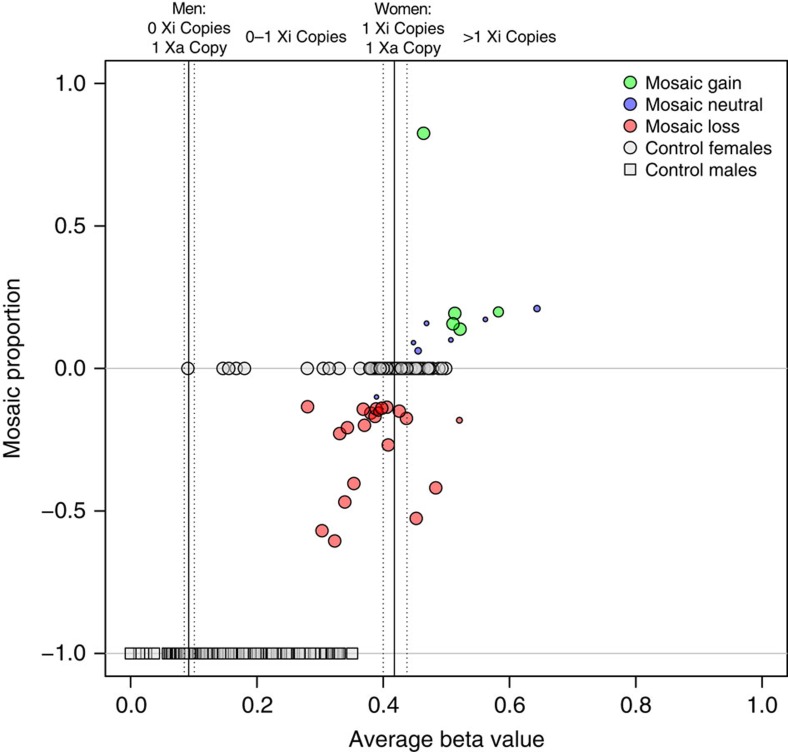
Chromosome X methylation beta values by estimated mosaic proportion. Average beta values (range: 0.0–1.0) indicate amount of methylation at a genomic locus where low values indicate hypomethylation and high values indicate hypermethylation. X methylation beta values are plotted for promoter probes spanning a mosaic X event and points are sized for the number of probes. The estimated mosaic proportion is calculated from the mosaic proportion estimate of the SNP microarrays and direction (positive versus negative) is determined from average probe beta *z*-scores. Control men (*N*=1,665) and women (*N*=136) are shown as light grey squares and circles. Mosaic females (*N*=48) are plotted as green, blue and red circles for mosaic gains, copy-neutral events and losses, respectively. Solid and dashed black lines are median and interquartile range for control men (left) and women (right) beta values.

To investigate the molecular basis of X-chromosome mosaicism, we used Illumina HumanMethylation450 microarray data for a subset of mosaic females with sufficient DNA to determine whether mosaic events are preferential for either the Xa or Xi. Established sex-specific differences in chromosome X promoter methylation^31^,^32^ provide an opportunity to determine whether the pattern of large structural mosaic events parallels what has previously been reported for analyses of somatic mutations in cancer, namely, events more likely occurring in the inactive X-chromosome^24^. After we completed a rigorous quality control process for methylation microarray data in a control population of 1,665 men and 136 women, probes in gene promoter sites on the X chromosome were extracted and filtered to focus analyses on a reference set of probes that were differentially methylated between men and women, as these are the locations that are inactivated on Xi (Supplementary Fig. 3)^31^,^32^. Methylation beta values for the resulting set of 1,888 probes were evaluated for differences from normal expected values in women (beta values greater than expected suggest mosaic gain of Xi and less than expected suggest mosaic loss of Xi) (Fig. 4). Of the 21 women with mosaic losses, 16 had evidence for a loss of the Xi chromosome. Similarly, all 5 women with mosaic gains had evidence suggesting a mosaic gain of Xi. For mosaic copy-neutral events, 6 women showed evidence for a loss of a portion of the Xa and a replacement with Xi and one woman showed evidence for a loss of a portion of Xi and a replacement with the Xa. Our combined data for mosaic gains and losses suggest that Xi is preferentially involved in mosaic copy-number changes, with Xi more commonly altered in mosaic losses and preferentially gained for mosaic gains (*P* value=0.002). Mosaic events on the X chromosome that do not follow this trend, particularly the five mosaic losses with evidence for a loss of the Xa, could represent normal variation, perhaps due to different DNA extraction techniques, noise in the methylation assay or statistical outliers. Alternatively, chromosome X events could occur early in female development, perhaps at a time that precedes X-inactivation, and thus X-inactivation could only occur in cells with more than one X chromosome.

## Discussion

Our analysis using SNP microarray intensities identified detectable mosaic events on the female X chromosome that occur at higher frequencies than mosaic events on the autosomes. We observed evidence that individual women with mosaic events of the X chromosome are also more likely to have mosaic events of the autosomes. Furthermore, X mosaic events are more likely to involve the inactive X chromosome than the active X chromosome, and thus might be phenotypically neutral. As with autosomal and Y mosaicism, X mosaicism increases with age.

For decades, it has been apparent that an appreciable fraction of paediatric developmental disorders are directly attributable to a spectrum of mosaic events (for example, from point mutations to large structural alterations) that can also influence clinical course^9^,^33^,^34^,^35^. Our data indicate that substantial numbers of adults also possess mosaic chromosomes in blood and buccal cells, suggesting the genome undergoes somatic alterations that either are generated later due to less efficient protective mechanisms or were perhaps tolerated from early age and subsequently expanded due to less efficient mechanisms for retaining genomic stability.

A limitation of our analysis is the low level of validation for partial chromosome copy-neutral events. Because of both the smaller event size and the need for log R ratio (LRR) baseline correction, our array-based detection algorithm together with qPCR-based validation yielded a low level of concordance. Further work is needed to improve the calling algorithms, which could also be accelerated by the analysis of larger samples sizes, ultimately leading to more precise measurement of mosaic X-chromosomal events.

It is striking that the frequency of large megabase mosaicism is higher in the inactive X as well as the Y chromosome compared with the autosomes. This higher frequency of mosaicism on sex chromosomes could be a reflection of less cell selection because the inactive X is transcriptionally inactive while the Y chromosome has the smallest number of genes. Future studies are needed to understand the mechanisms responsible for the generation and selection of these mosaic alterations in sex and autosomal chromosomes, which occur at different frequencies. In turn, insights into the underlying mechanisms as well as the differences in frequencies of large structural genetic mosaicism should provide an important foundation for understanding their contribution to health and chronic diseases^6^,^36^,^37^.

## Methods

### Study population

The data set was drawn from cancer GWAS of solid tumours performed at the National Cancer Institute Division of Cancer Epidemiology and Genetics and the Cancer Genome Research Laboratory. In total, peripheral leukocyte or buccal epithelial DNA was available for 20,878 solid tumour cancer cases and 17,425 cancer-free controls. DNA was genotyped on one or more commercially available Illumina Infinium Human SNP array (Hap300, Hap240, Hap550, Hap610, Hap660, Hap 1, Omni Express, Omni 1, Omni 2.5 and Omni 5). Quality control procedures were applied after genotyping and samples were clustered in batches to optimize accuracy and minimize batch effects. All GWAS studies were reviewed by the Institutional Review Board of the National Cancer Institute and those of the participating study centers. Informed consent was received for each study participant before study enrollment.

### Detection algorithm

BAF and LRR are two metrics used to detect mosaic events. BAF is a measure of allelic imbalance and used to quantify deviation of an individual's SNP genotype from expected AA, AB and BB genotype clusters. Contiguous runs of heterozygous SNPs with BAF values that deviate from the expected value of 0.5 are evidence for mosaicism. The LRR value of an individual's SNP is a proxy for copy number. LRR values are the log_2_ of the ratio of observed SNP intensity value to expected intensity value. LRR values greater than expected baseline LRR suggest copy gain and less than expected baseline LRR suggest copy loss. The expected baseline LRR was calculated from women within each clustering group based on the ratio of males and females in the original genotyping cluster group. All BAF and LRR values were calculated using methods described^38^ and renormalized as outlined previously^3^.

For female participants, BAF and LRR values were systematically scanned across the X chromosome. Chromosomes were segmented for mosaic events using circular binary segmentation (CBS) on BAF values with the BAF segmentation package^39^. Segments <2 Mb in size were filtered out to control the false-positive rate. Gaussian mixture models were fit to BAF bands to assign event type given the best-fitting model (2–4 Gaussian components). Event copy-number state was assigned based on LRR values with baselines adjusted for the number of men present within original genotyping cluster groups. For whole-chromosome mosaic X events, LRR deviations of 0.01 and −0.01 were used to classify events as gain and losses, respectively. For mosaic X events encompassing only a portion of the X chromosome, we chose a more conservative threshold of 0.05 and −0.05 for gains and losses due to greater LRR variation due to the reduced number of X probes that spanned the events. Mosaic proportions were estimated using deviation from the expected BAF given the LRR defined copy-number state. Further details are outlined in our prior work on autosomal mosaicism^3^.

### Quantitative PCR

qPCR assays were selected to determine copy-number status of 12 regions spanning the X chromosome by normalizing to an autosomal gene, RNase P, which is present in two copies in a diploid genome (Supplementary Table 4). One additional assay was run to validate the presence of the Y chromosome. According to Quant-iT PicoGreen dsDNA quantitation (Life Technologies, Grand Island, NY), 5 ng of sample DNA were transferred into LightCycler-compatible 384-well plates (Roche, Indianapolis, IN) in triplicate and dried down. Two internal standard curves were run separately in each plate, pooled gDNA samples of males and pooled gDNA samples of females, both with no detectable X chromosome loss/gain, and serially diluted to 6 concentrations. qPCR was performed using 5 μl reaction volumes consisting of: 2.5 μl of LightCycler 480 Probes Master Mix (Roche, Indianapolis, IN), 2.0 ul of MBG Water, 0.25 μl of 20 × TaqMan Copy Number Reference Assay, RNase P (Life Technologies, Grand Island, NY), and 0.25 μl of specific 20 × TaqMan Copy Number Assay (Life Technologies, Grand Island, NY). Thermal cycling was performed on a LightCycler 480 (Roche) where PCR conditions consisted of: 95 °C hold for 5 min, denature at 95 °C for 15 s, anneal at 60 °C for 30 s, with fluorescence data collection over 45 cycles. All experimental and control samples were assayed in triplicate on each plate, separately for all 12 individual target assays.

The LightCycler software (Release 1.5.0) was used for initial analysis of the raw data, utilizing the absolute quantification analysis with the second derivative maximum method and high-confidence detection algorithm, to yield a crossing threshold (Ct) for all replicates. The Ct for each assay was used to interpolate concentration of target and reference sequences using the standard curves. The ratio of target to reference was multiplied by 2 to determine the diploid amount of X chromosome in that region. The ratios of the 12 assays were then averaged to yield an overall X-chromosome signal ratio. Seventy-five normal copy-number controls were used to estimate normal probe ratio means and s.d. A value of 3 s.d. above the normal mean ratio was used as the threshold to call gains and a value of 3 s.d. below the normal mean ratio was the threshold for calling losses.

### Methylation arrays

After Quant-iT PicoGreen dsDNA quantitation (Life Technologies, Grand Island, NY), 1,000 ng of sample DNA were treated with sodium bisulfite using the EZ-96 DNA Methylation MagPrep Kit (Zymo Research, Irvine, CA) to convert unmethylated cytosine residues to uracils (detected as thymidines), leaving 5′-methylcytosines residues unaffected. Bisulfite-treated samples were denatured, neutralized and then whole-genome amplified, isothermally, to increase the amount of DNA template. The amplified product was enzymatically fragmented, precipitated and resuspended in hybridization buffer. Samples were hybridized overnight on Infinium HumanMethylation450 BeadChips (Illumina Inc., San Diego, CA), which allowed fragmented DNA to anneal to locus-specific 50mers (covalently linked to one of over 500,000 bead types). Single-base extension of oligonucleotides on the BeadChip, using the captured DNA as template, incorporated tagged nucleotides on the BeadChip, which were subsequently fluorophore labelled during staining. BeadChips were scanned by an Illumina iScan at two wavelengths to create image and intensity files. An internal control, a DNA sample from a lymphoblastoid cell line NA07057 (Coriell Cell Repositories, Camden, NJ), was utilized to confirm the efficiency of bisulfite conversion and subsequent methylation analysis.

Methylation beta values are indicators of site-specific methylation with a theoretical range from 0 to 1, where low values indicate hypomethylation and high values indicate hypermethylation. Raw beta intensity values were extracted for probes in promoter sites on the X chromosome and further filtered to include only probes that are differentially methylated between women (Xa/Xi) and men (Xa). A control sample of available men (*N*=1,665) and women (*N*=136) was used to determine expected beta value means and s.d. Using the RnBeads R library, promoter probes were selected that had mean beta values between 0.35 and 0.5 and s.d. <0.09 in women and mean beta values <0.15 and s.d. <0.05 in men (Supplementary Fig. 3). This left a total of 1,888 differentially methylated probes that spanned 212 promoter sites across the X chromosome for analysis. For each mosaic female, mean beta values and z-scores were calculated for all differentially methylated promoter probes that spanned detected mosaic X events in an effort to determine changes in methylation profiles and thus phase mosaic events to the Xa or Xi chromosomes. The mosaic proportions were calculated from SNP microarray per cent mosaicism values. Only X events spanning 5 or more promoter regions were used for the analysis.

### Statistical analysis

All statistical analyses were performed on a 64 bit Windows build of R 3.0.1 "Good Sport". Multivariate analyses used logistic regression models (glm procedure) with X mosaicism as the dependent variable and adjusted for age of DNA collection, study indicator variables, cancer status (case=1, control=0), and genetically inferred ancestry (%European, %African and %Asian) unless otherwise specified. Inferred ancestry proportions were estimated for each individual using reference populations from the HapMap project^40^ with the GLU software package (https://code.google.com/p/glu-genetics/) using the struct.admix module. Confidence intervals for plots are Wilson intervals. All reported *P* values are two-sided.

### Data availability

Original study data has been posted in dbGaP (http://www.ncbi.nlm.nih.gov/gap) under accession numbers phs000093.v2.p2, phs000336.v1.p1, phs000351.v1.p1, phs000361.v1.p1, phs000652.v1.p1, phs000716.v1.p1, phs000734.v1.p1, phs000396.v1.p1, phs000147.v2.p1, phs000346.v2.p1, phs000863.v1.p1 and phs000206.v5.p3. Data on called event features, location and individual characteristics are available in Supplementary Table 1. Methylation array beta values for events are presented in Supplementary Table 6 and raw data is posted in dbGaP under accession number phs001112.v1.p1. The methylation data has been deposited in dbGaP under accession code phs001112.v1.p1

## Additional information

**How to cite this article:** Machiela, M.J. *et al*. Female chromosome X mosaicism is age-related and preferentially affects the inactivated X chromosome. *Nat. Commun.* 7:11843 doi: 10.1038/ncomms11843 (2016).

## Supplementary Material

Supplementary Information Supplementary Figures 1-3 and Supplementary Tables 1-6.

## Figures and Tables

**Table 1:** Chromosomal arm location of detected mosaic autosomal and X events.

	Autosomes	X chromosome
Interstitial	148 (34.4%)	46 (37.1%)
Spans centromere	17 (4.0%)	2 (1.6%)
Telomeric p	95 (22.1%)	4(3.2%)
Telomeric q	148 (34.4%)	12 (9.7%)
Whole	22 (5.1%)	60 (48.4%)
	430 (100%)	124 (100%)
