# Supplementary material for: Female chromosome X mosaicism is age-related and preferentially affects the inactivated X chromosome
Source: Nat Commun. 2016 Jun 13;7:11843. doi: 10.1038/ncomms11843 (PMC4909985; doi:10.1038/ncomms11843)
Supplement: Supplementary Information — Supplementary Figures 1-3 and Supplementary Tables 1-6. [file ncomms11843-s1.pdf]

Supplementary Figure 1. Distribution of detected mosaic events by copy number state for (A) the autosomes and (B) the X chromosome. Labels indicate event copy number state and numbers in parentheses are counts for total number of detected events.

(A)

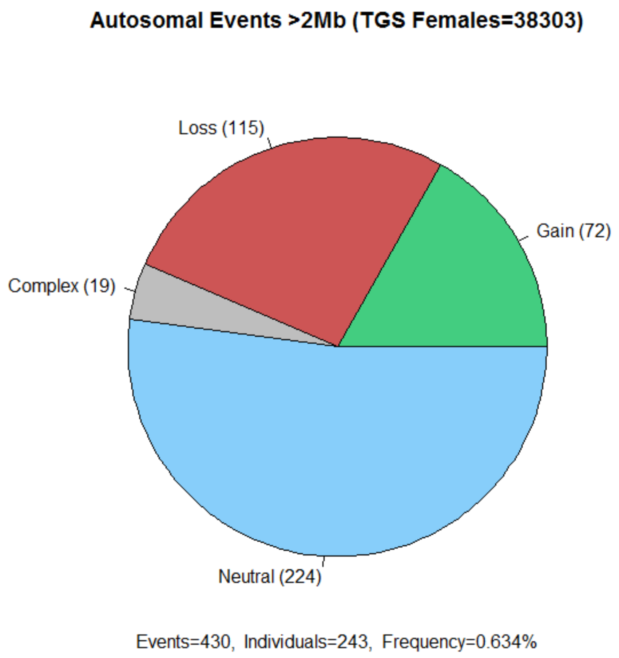

(B)

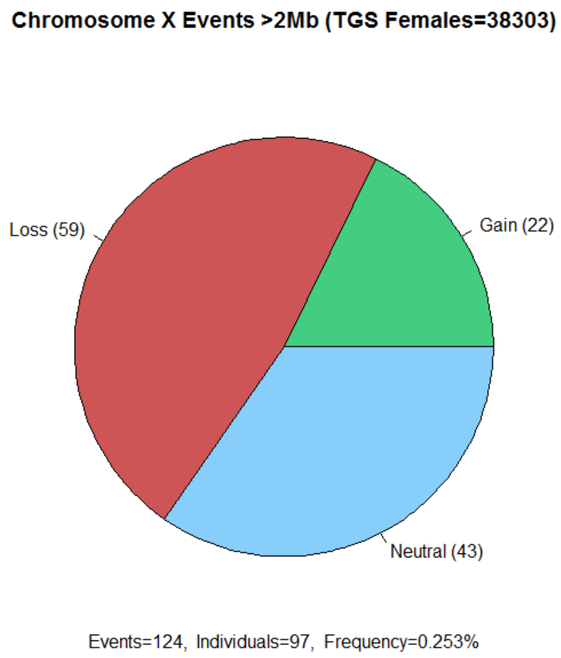

Supplementary Figure 2. Distribution of mosaic proportions for detected mosaic X events in comparison to detected autosomal mosaic events. P-value tests for a difference in distributional means.

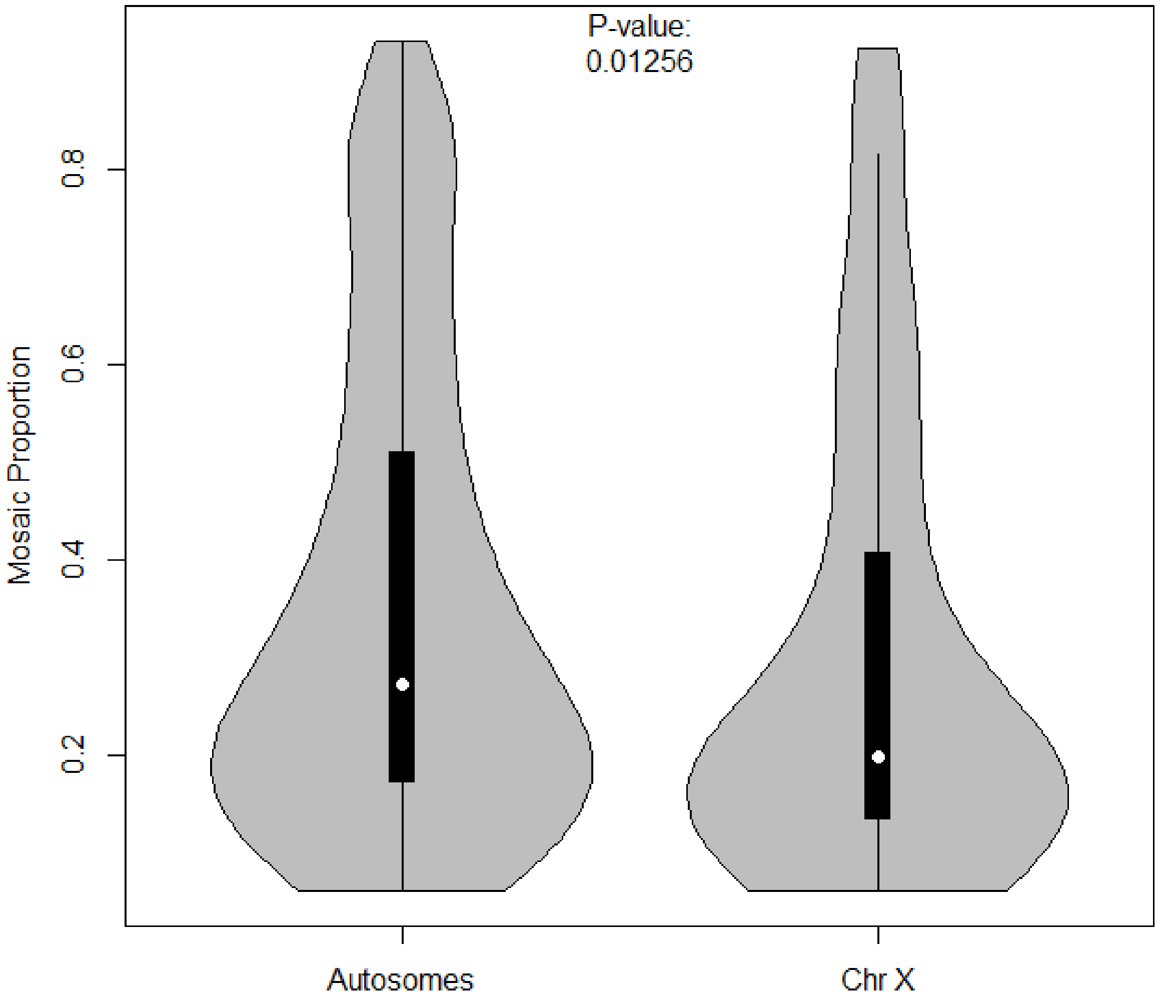

Supplementary Figure 3. Mean beta values and standard deviations for methylation probe promoter regions for the Illumina Methyl450 array in 1,665 control men and 136 control women. (A) Promoter regions selected in women had mean beta values between 0.35 and 0.5 and standard deviations less than 0.09. (B) The filtered promoter probes in women were investigated in men and further filtered to only include promoter probe regions with mean beta values less than 0.15 and standard deviations less than 0.05. In total, 1,888 differentially methylated probes that spanned 212 promoter sites across the X chromosome were included in subsequent analyses.

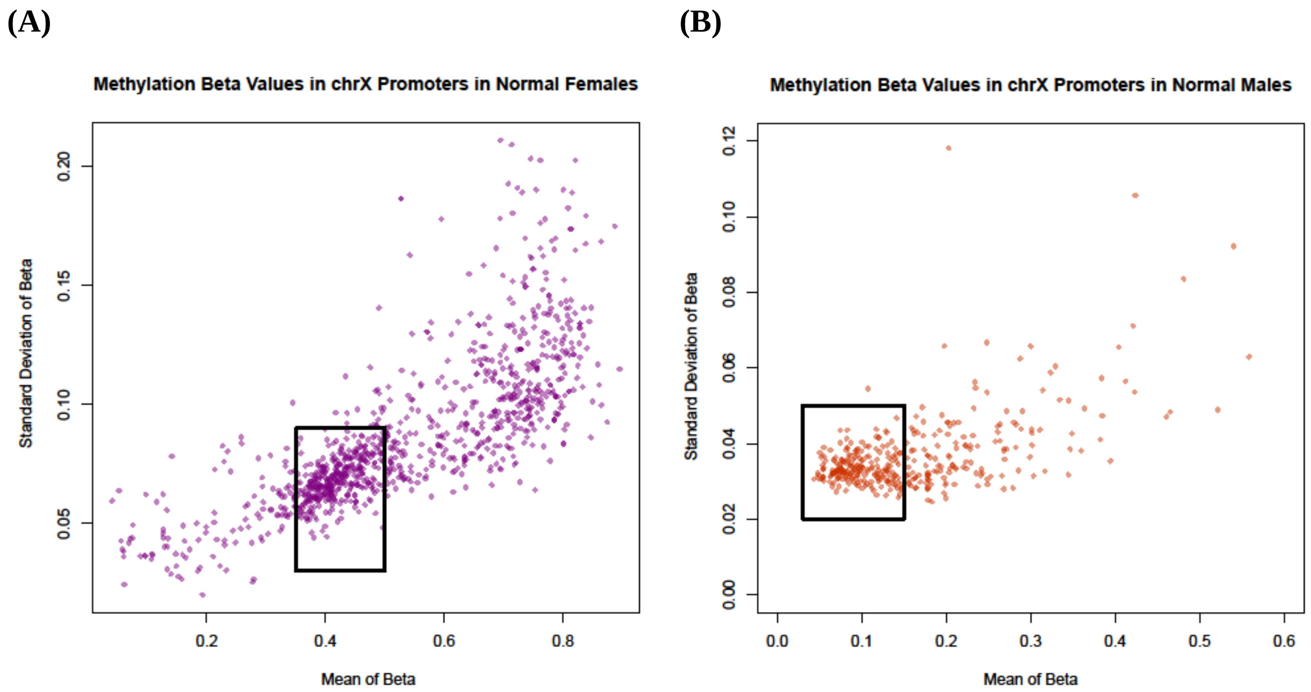

Supplementary Table 1. Locations and individual characteristics of detected chromosome X events.

ID	Chr	Start	End	Size	Probes	State	Mosaic Percent	Sex	Age DNA	Cancer
1	X	1,553	154,582,606	154,581,053	13,331	Loss	0.511	Female	NA	Gastric
2	X	31,108,125	33,712,571	2,604,446	642	Neutral	0.510	Female	71	Stomach
3	X	132,234,466	154,582,606	22,348,140	2,634	Neutral	0.092	Female	65	Cancer-free
4	X	1,553	154,582,606	154,581,053	13,906	Loss	0.689	Female	NA	Lung
5	X	67,840,460	154,446,085	86,605,625	6,921	Loss	0.904	Female	39	Lung
6	X	38,541,601	41,214,219	2,672,618	346	Neutral	0.137	Female	61	Breast
7	X	45,000,000	134,901,714	89,901,714	20,916	Gain	0.198	Female	71	Cancer-free
8	X	9,120,838	20,667,813	11,546,975	1,345	Neutral	0.124	Female	54	Cancer-free
8	X	37,332,880	47,772,472	10,439,592	1,044	Neutral	0.134	Female	54	Cancer-free
8	X	117,279,682	119,548,573	2,268,891	231	Neutral	0.250	Female	54	Cancer-free
8	X	152,290,349	154,553,040	2,262,691	155	Neutral	0.350	Female	54	Cancer-free
9	X	140,204,533	146,339,953	6,135,420	832	Neutral	0.118	Female	66	Cancer-free
9	X	22,000,000	34,450,748	12,450,748	1,886	Neutral	0.119	Female	66	Cancer-free
9	X	78,809,020	99,500,000	20,690,980	1,429	Neutral	0.119	Female	66	Cancer-free
9	X	114,658,108	128,000,000	13,341,892	1,338	Neutral	0.119	Female	66	Cancer-free
10	X	1,553	154,582,606	154,581,053	12,246	Loss	0.157	Female	63	Lung
11	X	111,317,946	152,522,337	41,204,391	4,341	Loss	0.182	Female	46	Lung
12	X	1,553	154,579,620	154,578,067	13,311	Loss	0.134	Female	66	Breast
13	X	9,402,279	20,000,000	10,597,721	3,606	Neutral	0.172	Female	67	Cancer-free
13	X	37,595,757	48,938,568	11,342,811	3,513	Neutral	0.213	Female	67	Cancer-free
14	X	99,148,316	129,076,960	29,928,644	3,502	Loss	0.660	Female	NA	Cancer-free
15	X	12,859,359	22,057,972	9,198,613	805	Neutral	0.100	Female	60	Breast
15	X	36,000,000	48,000,000	12,000,000	1,124	Neutral	0.100	Female	60	Breast
16	X	1,553	155,260,560	155,259,007	13,282	Gain	0.825	Female	56	Cancer-free
17	X	9,225,116	22,000,000	12,774,884	1,835	Neutral	0.210	Female	NA	Endometrial
17	X	35,000,000	56,000,000	21,000,000	2,285	Gain	0.649	Female	NA	Endometrial
18	X	1,553	155,260,560	155,259,007	39,903	Loss	0.351	Female	50	Cancer-free
19	X	2,500,000	154,582,606	152,082,606	17,998	Loss	0.383	Female	NA	Endometrial
20	X	1,553	155,260,560	155,259,007	38,666	Loss	0.209	Female	80	Cancer-free
21	X	1,553	155,260,560	155,259,007	13,287	Loss	0.168	Female	63	Multiple
22	X	1,553	155,260,560	155,259,007	13,235	Gain	0.839	Female	62	Glioma
23	X	1,553	154,582,606	154,581,053	12,787	Gain	0.206	Female	52	Lung
24	X	38,482,237	41,458,510	2,976,273	374	Neutral	0.138	Female	77	Breast
25	X	1,553	154,579,620	154,578,067	40,102	Loss	0.179	Female	77	Lung
26	X	37,332,880	41,458,510	4,125,630	514	Neutral	0.091	Female	73	Breast
26	X	15,000,000	21,000,000	6,000,000	499	Neutral	0.097	Female	73	Breast
27	X	3,427,253	154,443,832	151,016,579	13,101	Loss	0.353	Female	71	Cancer-free
28	X	115,322,150	154,090,810	38,768,660	5,681	Neutral	0.077	Female	NA	Cancer-free
29	X	2,500,000	154,582,606	152,082,606	17,892	Gain	0.277	Female	NA	Endometrial
30	X	1,553	154,582,606	154,581,053	13,338	Loss	0.348	Female	NA	Bladder
31	X	2,500,000	154,582,606	152,082,606	18,098	Loss	0.643	Female	NA	Cancer-free

32	X	2,724,756	154,582,606	151,857,850	14,057	Gain	0.866	Female	49	Lung
33	X	2,735,883	154,582,606	151,846,723	39,632	Loss	0.155	Female	67	Lung
34	X	9,120,838	20,473,147	11,352,309	1,321	Neutral	0.158	Female	64	Breast
34	X	37,332,880	47,772,472	10,439,592	1,045	Neutral	0.178	Female	64	Breast
34	X	117,279,682	119,548,573	2,268,891	233	Neutral	0.178	Female	64	Breast
35	X	1,553	154,582,606	154,581,053	13,324	Loss	0.719	Female	NA	Unknown
36	X	125,683,733	154,138,131	28,454,398	2,966	Gain	0.245	Female	38	Lung
37	X	1,553	154,582,606	154,581,053	13,336	Loss	0.307	Female	NA	Unknown
38	X	30,548,244	33,300,070	2,751,826	665	Loss	0.519	Female	74	Esophagus
39	X	27,015,006	32,083,817	5,068,811	766	Loss	0.182	Female	56	Lung
39	X	2,835,403	16,000,000	6,343,174	764	Gain	0.222	Female	56	Lung
40	X	2,500,000	154,582,606	152,082,606	17,860	Loss	0.230	Female	NA	Endometrial
41	X	1,553	154,582,606	154,581,053	12,972	Loss	0.324	Female	70	Lung
42	X	1,553	154,582,606	154,581,053	12,551	Gain	0.880	Female	68	Lung
43	X	143,004,816	145,031,290	2,026,474	564	Loss	0.487	Female	70	Cancer-free
44	X	1,553	155,260,560	155,259,007	13,260	Loss	0.168	Female	62	Esophagus
45	X	36,000,000	50,000,000	14,000,000	1,151	Neutral	0.095	Female	64	Lung
45	X	111,478,662	154,712,424	43,233,762	4,337	Neutral	0.198	Female	64	Lung
45	X	2,787,560	15,086,954	12,299,394	1,554	Neutral	0.225	Female	64	Lung
45	X	92,000,000	96,000,000	4,000,000	365	Gain	0.242	Female	64	Lung
46	X	1,553	154,582,606	154,581,053	13,342	Loss	0.663	Female	NA	Bladder
47	X	1,553	154,579,620	154,578,067	39,220	Loss	0.143	Female	68	Cancer-free
48	X	1,553	154,582,606	154,581,053	13,332	Gain	0.157	Female	NA	Gastric
49	X	1,553	155,260,560	155,259,007	13,280	Loss	0.434	Female	77	Cancer-free
50	X	2,519	72,267,780	72,265,261	6,316	Gain	0.129	Female	68	Breast
50	X	72,267,781	155,260,560	82,992,779	6,875	Loss	0.214	Female	68	Breast
51	X	94,926,466	127,956,023	30,029,557	2,457	Neutral	0.097	Female	60	Lung
51	X	135,049,024	152,850,324	17,801,300	2,167	Loss	0.148	Female	60	Lung
52	X	148,371,055	152,616,777	4,245,722	560	Neutral	0.080	Female	70	Cancer-free
52	X	37,332,880	48,895,426	11,562,546	1,093	Neutral	0.090	Female	70	Cancer-free
53	X	2,500,000	154,582,606	152,082,606	17,821	Loss	0.253	Female	NA	Endometrial
54	X	38,551,737	41,483,959	2,932,222	908	Neutral	0.100	Female	77	Lung
55	X	37,537,457	48,000,000	10,462,543	1,479	Neutral	0.162	Female	NA	Cancer-free
56	X	85,385,491	154,477,684	69,092,193	19,160	Neutral	0.079	Female	75	Cancer-free
57	X	21,691,798	36,511,216	14,819,418	2,056	Neutral	0.100	Female	49	Lung
57	X	77,009,442	98,330,836	21,321,394	1,449	Neutral	0.100	Female	49	Lung
57	X	111,316,727	125,023,781	13,707,054	1,367	Neutral	0.100	Female	49	Lung
57	X	140,000,000	148,000,000	8,000,000	1,038	Neutral	0.100	Female	49	Lung
58	X	1,553	154,582,606	154,581,053	12,255	Loss	0.126	Female	56	Cancer-free
59	X	108,000,000	115,904,104	7,904,104	821	Loss	0.923	Female	NA	Cancer-free
60	X	2,500,000	154,582,606	152,082,606	17,884	Loss	0.887	Female	NA	Endometrial
61	X	1,553	155,260,560	155,259,007	13,072	Loss	0.625	Female	NA	Cancer-free
62	X	1,553	155,260,560	155,259,007	13,288	Gain	0.821	Female	54	Cancer-free
63	X	2,500,000	154,582,606	152,082,606	17,830	Loss	0.218	Female	NA	Cancer-free

64	X	1,553	154,579,620	154,578,067	13,257	Loss	0.136	Female	65	Breast
65	X	12,000,000	22,078,734	10,078,734	3,246	Neutral	0.099	Female	62	Cancer-free
65	X	128,264,436	134,901,714	6,637,278	1,943	Neutral	0.110	Female	62	Cancer-free
65	X	38,056,705	75,228,249	37,171,544	8,227	Neutral	0.124	Female	62	Cancer-free
65	X	99,732,340	109,706,724	9,974,384	3,049	Neutral	0.128	Female	62	Cancer-free
65	X	117,331,631	119,971,453	2,639,822	826	Neutral	0.147	Female	62	Cancer-free
66	X	99,573,032	114,378,172	14,805,140	865	Gain	0.862	Female	58	Lung
67	X	1,553	154,582,606	154,581,053	12,783	Gain	0.653	Female	67	Lung
68	X	1,553	155,260,560	155,259,007	14,971	Loss	0.569	Female	67	Lung
69	X	28,343,230	31,443,128	3,099,898	436	Loss	0.620	Female	NA	Cancer-free
70	X	36,511,216	55,616,996	19,105,780	1,646	Neutral	0.095	Female	72	Cancer-free
71	X	2,787,560	154,582,606	151,795,046	13,813	Loss	0.185	Female	78	Cancer-free
72	X	39,483,015	53,636,899	14,153,884	1,156	Loss	0.182	Female	68	Lung
73	X	1,553	155,260,560	155,259,007	13,736	Loss	0.605	Female	44.	Breast
74	X	1,553	155,260,560	155,259,007	13,729	Loss	0.199	Female	65	Breast
75	X	1,553	155,260,560	155,259,007	13,632	Loss	0.369	Female	74	Lung
76	X	2,750,681	154,450,686	151,700,005	14,954	Loss	0.451	Female	62	Cancer-free
77	X	1,553	155,260,560	155,259,007	14,976	Gain	0.157	Female	60	Bladder
78	X	1,553	155,260,560	155,259,007	15,012	Gain	0.138	Female	80	Lung
79	X	1,553	155,260,560	155,259,007	14,917	Loss	0.175	Female	80	Lung
80	X	1,553	155,260,560	155,259,007	13,773	Loss	0.142	Female	71	Pancreas
81	X	1,553	155,260,560	155,259,007	14,956	Loss	0.250	Female	54	Pancreas
82	X	1,553	155,260,560	155,259,007	15,044	Loss	0.404	Female	79	Cancer-free
83	X	1,553	155,260,560	155,259,007	14,956	Loss	0.268	Female	78	Lung
84	X	1,553	155,260,560	155,259,007	15,041	Loss	0.526	Female	72	Cancer-free
85	X	1,553	155,260,560	155,259,007	13,365	Gain	0.638	Female	54	Cancer-free
86	X	12,500,000	50,000,000	37,500,000	4,470	Neutral	0.062	Female	66	Lung
87	X	33,450,778	125,592,155	92,141,377	7,377	Loss	0.152	Female	73	Cancer-free
87	X	125,592,156	153,890,083	28,297,927	3,249	Gain	0.552	Female	73	Cancer-free
88	X	1,553	155,260,560	155,259,007	13,363	Loss	0.229	Female	66	Glioma
89	X	1,553	155,260,560	155,259,007	14,963	Loss	0.373	Female	82	Cancer-free
90	X	1,553	155,260,560	155,259,007	15,034	Gain	0.193	Female	72	Bladder
91	X	1,553	155,260,560	155,259,007	33,108	Loss	0.469	Female	70	Bladder
92	X	1,553	155,260,560	155,259,007	14,989	Loss	0.208	Female	68	Kidney
93	X	1,553	154,579,620	154,578,067	14,976	Loss	0.150	Female	71	Cancer-free
94	X	77,414,128	153,886,968	76,472,840	6,995	Gain	0.213	Female	74	Cancer-free
95	X	1,553	155,260,560	155,259,007	14,999	Loss	0.419	Female	82	Lung
96	X	2,750,681	154,582,606	151,831,925	15,019	Loss	0.170	Female	64	Cancer-free
97	X	1,553	154,579,620	154,578,067	14,972	Loss	0.140	Female	68	Cancer-free

Supplementary Table 2. Mosaic X events by contributing study.

	No X Mosaicism	X Mosaicism	Total
**AHS**	20	1	21
**AustraliaCancerStudy**	29	0	29
**Beijing**	791	0	791
**Brazil**	51	0	51
**CeRePP**	38	1	39
**CLUEII**	74	1	75
**CNULCS**	1,080	4	1,084
**COG**	265	0	265
**CONN**	1,031	2	1,033
**CONN-OLD**	66	0	66
**CPSII**	1,618	13	1,631
**EAGLE**	860	1	861
**EPIC**	801	2	803
**FBCS**	30	1	31
**FHCRC**	1,401	3	1,404
**FTCS**	69	0	69
**Fudan**	584	0	584
**GELAC**	1,198	3	1,201
**Gliogene**	145	1	146
**GROUPHEALTH**	272	1	273
**Guangdong**	711	1	712
**HERPACC**	633	0	633
**HKLymphoma**	378	1	379
**HPFS**	1	1	2
**Interphone**	269	0	269
**Italy**	21	0	21
**JHU**	235	0	235
**Korea-Univ**	103	0	103
**Kyungpook-Univ**	221	1	222
**MAYO**	579	0	579
**MCCS**	54	0	54
**MDA**	222	0	222
**MDA_Amos_Spitz_Wu**	354	1	355
**MDA_McNeil**	595	0	595
**MEC**	641	1	642
**Melbourne**	23	0	23
**MSKCC**	149	0	149
**NCC**	278	0	278
**NCI_BTS**	366	1	367
**NCI-UMD**	277	2	279
**NEBCS**	296	1	297

**NEET**	593	3	596
**NHS**	3,397	3	3,400
**NHS2**	546	1	547
**NIOSH_AGCC**	357	1	358
**NITC**	606	3	609
**NSHDS**	477	0	477
**NYU-WHS**	33	0	33
**Osteosarcoma**	37	0	37
**PamplonaCUN**	37	0	37
**PBCS**	1,140	7	1,147
**PECC**	487	0	487
**PeterMac**	3	0	3
**PLCO**	2,804	13	2,817
**POCC**	260	0	260
**Poland**	155	0	155
**SBCS**	261	1	262
**SCCS**	203	1	204
**Shenyang**	1,007	5	1,012
**SHNX**	1,134	0	1,134
**SING**	129	0	129
**SINGAPORE**	493	0	493
**SMWHS**	124	0	124
**SNU**	162	0	162
**SNUH**	44	0	44
**SNUPM**	83	0	83
**Sweden**	31	0	31
**SWHS**	619	0	619
**Taiwan**	1,031	1	1,032
**Tianjin**	478	1	479
**TORONTO**	260	1	261
**UCL_UK**	15	0	15
**UCSF**	750	3	753
**USMulticenter**	8	0	8
**USRC**	773	2	775
**WayneState**	355	1	356
**WHI**	976	3	979
**WHS**	57	0	57
**Wuhan**	63	0	63
**Xuan-Wei**	1,155	2	1,157
**YALE**	234	1	235
**Total**	**38,206**	**97**	**38,303**

Supplementary Table 3. Chromosomal arm location by copy number state.

	Gain		Loss		Neutral		Total	
interstitial	3	6.5%	8	17.4%	35	76.1%	46	100%
spans centromere	1	50.0%	0	0.0%	1	50.0%	2	100%
telomeric p	2	50.0%	0	0.0%	2	50.0%	4	100%
telomeric q	3	25.0%	4	33.3%	5	41.7%	12	100%
whole	13	21.7%	47	78.3%	0	0.0%	60	100%
	22	17.7%	59	47.6%	43	34.7%	124	100%

Supplementary Table 4. Validation qPCR probes and locations.

SNP or GENE Symbol	Gene Name	Chr	Location (NCBI Build 37)	Life Assay ID	NCBI Location	Cytoband Location	Assay Location	Amplicon Length
XG	Xg blood group	X	2703859	Hs04100849_cn	ChrX:2703633-2805662	Xp22.33c	Within Intron 4	82
PHEX	phosphate regulating endopeptidase homolog; X-linked	X	22053233	Hs05679343_cn	ChrX:22050921-22266478	Xp22.11b	Within Intron 1	86
CXorf22	chromosome X open reading frame 22	X	35937936	Hs01859438_cn	ChrX:35937851-36008269	Xp21.1b	Within Exon 1	88
KDM6A	lysine (K)-specific demethylase 6A	X	44736584	Hs05632292_cn	ChrX:44732423-44971847	Xp11.3b	Within Intron 2	98
CXorf65	chromosome X open reading frame 65	X	70325895	Hs00598842_cn	ChrX:69640535-71108033	Xq13.1d	Within Exon 3	80
PCDH11X	protocadherin 11 X-linked	X	91127540	Hs05604144_cn	ChrX:91099385-91199647	Xq21.31e	Within Intron 4	104
CXorf57	chromosome X open reading frame 57	X	105922389	Hs02805049_cn	ChrX:104949928-106367102	Xq22.3b	Within Exon 14	110
CXorf61	chromosome X open reading frame 61	X	115593024	Hs01583891_cn	ChrX:115589779-115624958	Xq23e	Within Exon 2	101
CXorf64	chromosome X open reading frame 64	X	125954155	Hs04097898_cn	ChrX:125930015-125965957	Xq25f	Within Intron 1	94
RBMX	RNA binding motif protein; X-linked	X	135952571	Hs05654346_cn	ChrX:135951353-135962939	Xq26.3c	Overlaps Intron 10 - Exon 11	107
SPANXN3	SPANX family; member N3	X	142596950	Hs02664978_cn	ChrX:142594742-142798873	Xq27.3a	Overlaps Intron 1 - Exon 2	109
PLXNA3	plexin A3	X	153701896	Hs01014974_cn	ChrX:153348431-153829693	Xq28g	Within Exon 33	103
NLGN4Y	neuroligin 4; Y-linked	Y	16680289	Hs05707486_cn	ChrY:16634488-16955848	Yq11.221c	Within Intron 4	104

Supplementary Table 5. Validation results from qPCR and concordance rates with array genotyping.

Whole Chromosome

		qPCR					
		**Loss**	**Neutral**	**Gain**	**Failed**		**Concordance**
**GWAS Array**	**Loss**	12	2	1	0	15	**80.00%**
	**Neutral**	0	0	0	0	0	**NA**
	**Gain**	0	0	3	0	3	**100.00%**
		12	2	4	0	18	**83.33%**

Partial Chromosome

		qPCR					
		**Loss**	**Neutral**	**Gain**	**Failed**		**Concordance**
**GWAS Array**	**Loss**	0	1	1	0	2	**0.00%**
	**Neutral**	0	1	2	2	5	**20.00%**
	**Gain**	0	0	1	0	1	**100.00%**
		0	2	4	2	8	**25.00%**

Supplementary Table 6. Average beta and percent mosaic values for methylation samples.

Sample	Copy Number State[1]	Average Beta[2]	Percent Mosaic[1]
1	Loss	0.280	0.134
2	Loss	0.303	0.569
3	Loss	0.323	0.605
4	Loss	0.331	0.229
5	Loss	0.339	0.469
6	Loss	0.343	0.208
7	Loss	0.354	0.404
8	Loss	0.368	0.143
9	Loss	0.370	0.199
10	Loss	0.380	0.157
11	Loss	0.387	0.168
12	Loss	0.389	0.142
13	Neutral	0.389	0.100
14	Loss	0.392	0.152
15	Loss	0.398	0.140
16	Loss	0.406	0.136
17	Loss	0.408	0.268
18	Loss	0.422	0.519
19	Loss	0.425	0.150
20	Loss	0.437	0.175
21	Neutral	0.448	0.091
22	Loss	0.452	0.526
23	Neutral	0.455	0.062
24	Gain	0.464	0.825
25	Neutral	0.469	0.158
26	Loss	0.483	0.419
27	Neutral	0.486	0.118
28	Neutral	0.501	0.138
29	Neutral	0.507	0.100
30	Neutral	0.510	0.100
31	Gain	0.510	0.157
32	Gain	0.513	0.193
33	Loss	0.521	0.182
34	Gain	0.522	0.138
35	Neutral	0.562	0.172
36	Gain	0.582	0.198
37	Neutral	0.613	0.137
38	Neutral	0.643	0.210

(1) Copy number state and percent mosaicism determined from SNP genotyping arrays

(2) Beta value from Illumina HumanMethylation450 arrays
